# Supplementary material for: Virus-like Particles of Nodavirus Displaying the Receptor Binding Domain of SARS-CoV-2 Spike Protein: A Potential VLP-Based COVID-19 Vaccine
Source: Int J Mol Sci. 2023 Feb 23;24(5):4398. doi: 10.3390/ijms24054398 (PMC10001971; doi:10.3390/ijms24054398)
Supplement: Supplementary file 1 [file ijms-24-04398-s001.zip › ijms-2107693-supplementary.pdf]

SUPPLEMENTAL MATERIALS

Virus-like Particles of Nodavirus Displaying the Receptor Binding Domain of SARS-CoV-2 Spike Protein: A potential VLP-based COVID-19 Vaccine

Kiven Kumar [1], Wen Siang Tan [2], Siti Suri Arshad [3], Kok Lian Ho [1*]

[1]Department of Pathology, Faculty of Medicine and Health Sciences, Universiti Putra Malaysia, 43400 UPM Serdang, Selangor, Malaysia;
[2]Department of Microbiology, Faculty of Biotechnology and Biomolecular Sciences, Universiti Putra Malaysia, 43400 UPM Serdang, Selangor, Malaysia;
[3]Department of Veterinary Pathology and Microbiology, Faculty of Veterinary Medicine, Universiti Putra Malaysia, 43400 UPM Serdang, Selangor, Malaysia;
* Correspondence: klho@upm.edu.my; Tel.: +603-9769-2729

Figure S1

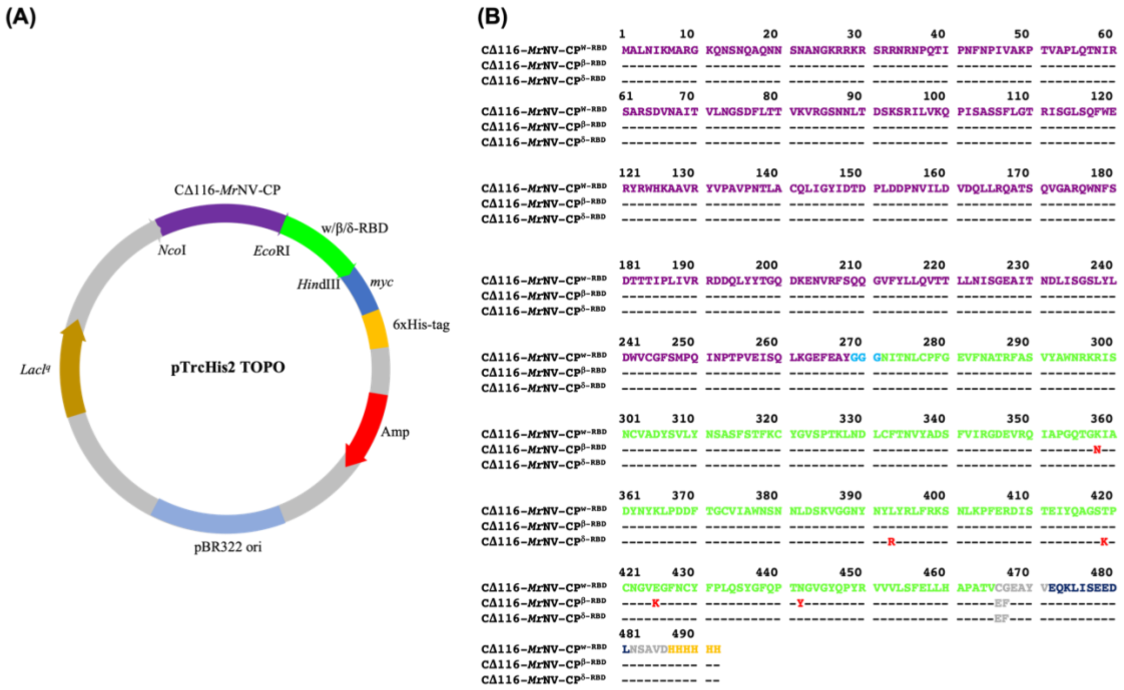

Figure S1. Construction of recombinant plasmids encoding the fusion protein of the protruding domain truncated *Macrobrachium rosenbergii* nodavirus capsid protein (CΔ116-*Mr*NV-CP) and the receptor binding domain (RBD) of severe acute respiratory syndrome coronavirus 2 spike (S) protein. A) A map of recombinant plasmid harboring the coding sequence of CΔ116-*Mr*NV-CP (purple) fused with the coding sequence of the β/δ-RBD (green) at its 3′ end, followed by the *myc* (dark blue) and 6xHis-tag (yellow) nucleotide sequences. B) Sequence alignment of the primary amino acid sequences of the fusion proteins CΔ116-*Mr*NV-CP$^{w\text{-}RBD}$ (derived from ancestral Wuhan strain), CΔ116-*Mr*NV-CP$^{\beta\text{-}RBD}$ and CΔ116-*Mr*NV-CP$^{\delta\text{-}RBD}$ is colored as described above. Amino acid residues colored in red represent variations as compared with the original Wuhan strain. Dashes indicate amino acids identical to the amino acid sequence of CΔ116-*Mr*NV-CP$^{w\text{-}RBD}$.
